# Reviews and Book Notices

**Published:** 1877-10

**Authors:** 


### Reviews and Book Notices.

Cyclopædia oe the Practice of Medicine. Edited by *H. Von Ziemssen*, Vol. XII. Diseases of the Nervous System. By *Prof. Nothnagel* of Jena, *Prof. E. Hitzig*, of Zurich, *Prof. F. Obernier*, of Bonn, *Prof. O. Heubner*, of Leipzig, and *Prof. G. Huguenin*, of Zurich. *William Wood & Co.*, New York.

Prof. Nothnagel's monograph, considers Anæmia, Hyperæmia, Hemorrhage, Thrombosis, and Embolism of the Brain, and treats most ably of the morbid states in question. The translation is by Dr. James J. Putnam, and is creditably executed.

Obernier. Takes up the obscure and difficult study of tumors of the brain and its membranes, and has collected an amount of data that the casual student would not have supposed existed on the subject, and while leaving much to be desired, and much that remains indefinite, has made a positive contribution to our knowledge of this class of cases. Translated by Dr. Henry R. Swanzy.

Heubner. Has studied exhaustively the effects of Syphilis upon the brain, its membranes and the peripheral nerves. No specially new additions have been made to our previous knowledge of the subject, but he has collected the facts which were hitherto widely scattered, and has earned the thanks of the profession for a clear, lucid statement, in compact form, of our possessions and resources for treatment of these diseases. Translated by Robert T. Edes, M. D.

Huguenin. Takes up the study of the chronic and acute inflammations of the brain, and has given on this important group one of the most valuable monographs yet published. His style is clear, and statements supported by a wide experience, while his deductions logically drawn, lead us at once to attach great importance to his opinions. It is the longest and most elaborate of the five monographs constituting the volume. It is translated by Drs. Bradford, Shattuck and Cutler.

Hitzig's Memoirs on hypertrophy and atrophy of the brain are of interest, as is everything coming from the pen of this distinguished observer. After a careful study of the pathology of these diseases, he advises what almost all alienists would endorse—the early removal of the patient to a well conducted asylum, furnished with large grounds. His first requisite in treatment is *rest* under continuous skilled oversight; *fresh air*, good *nourishment*. His therapeutics do not differ essentially from that usually adopted in the treatment of the better class of American institutions. The memoirs are well translated by Dr. S. G. Webber.

CYCLOPÆDIA OF THE PRACTICE OF MEDICINE: Edited by *Dr. H. Von Ziemssen*, Professor of Clinical Medicine in Munich, Bavaria. Vol. VII. Diseases of the Chylopoetic System, together with the Chapters on Diseases of the Naso-pharyngeal Cavity and Pharynx, Laryngitis Phlegmenosa, Perichondritis Laryngea, Ulcerations and Tumors and Neuroses of the Larynx, by *Prof. Wendt*, of Leipzig; *Prof. W. Leube*, of Jena; *Dr. O. Leichtenstern*, of Tuebingen; *Prof. Heller*, of Kiel; *Prof. Von Ziemssen*, of Munich, and *Dr. A. Steffen*, of Steffn: Wm. Wood & Co. 1876. Octavo; pp. 1,046.

The first article is an exhaustive one, by the late Dr. Herman Wendt, of Leipzig, on diseases of the naso-pharyngeal cavity and pharynx. Dr. W. discusses the general anatomy of the region, its participation in respiration, in deglutition, and in speech, and its relations to the ear. Then he reviews the general symptomatology, diagnosis and treatment of the various diseases of the locality. The article, as a whole, strikes us favorably, not so much because of its originality as of the painstaking and laborious examination of authorities, and judicious selection displayed in the compilation. We take exception, emphatically, to the recommendation he makes of the nasal douche, "as a valuable and, with ordinary care, harmless instrument in the treatment of nasal catarrh," and can only account for his freedom from accident in its use, by supposing his practical experience with it was not extensive.

The article on diseases of the stomach and intestines, by Prof. Leube, has an admirable introductory chapter on the anatomical and physiological relations and functions of the stomach.

The chapter on ulcer of the stomach is well worth careful study; it is comprehensive, thorough and the therapy is scientific.

The article by Prof. Leichtenstern, on constrictions, occlusions and displacements of the intestines, is full but heavy and uninteresting, and is mainly a compilation, but as such is very valuable.

The article on intestinal parasites is a success, so far as classification, etiology, general pathology and diagnosis are concerned, but the treatment proposed for some varieties seems to be unnecessarily elaborate; for example, the method recommended for the treatment of oxyuris vermicularis, is washing out the large intestine with a solution of soap—an enema tube eighteen inches long and a glass funnel being used for the purpose. The patient is placed in various positions on his hands and knees, on his belly, and on his back, and with thighs well flexed on the abdomen; three or four quarts of water being thus introduced, under a moderate pressure.

About one-fifth of this volume is devoted to diseases of the larynx. The author, Prof. H. Von Ziemssen, from an extended experience and careful study, has been enabled to furnish a most excellent chapter upon these diseases. He has carefully described plegmonous laryngitis, inflammations of the perichondrium, ulcerations and tumors of the larynx, and the various neuroses to which this organ is subject. The article is written with the clearness and precision which mark the productions of this author, and is amply illustrated with wood cuts, so that those not familiar with these subjects could hardly do better than read this chapter.

An anatomico-physiological introduction precedes the article on the neuroses, which, by the aid of three or four wood cuts, gives a clear idea of the functions of the muscles and nerves of the larynx, as at present understood. As students of anatomy often neglect this subject, we think the majority of physicians would find this of very great interest.

In the treatment of syphilitic affections of the larynx, the author urges the necessity of active treatment in the endeavor to check the progress of the disease as soon as possible.

He greatly prefers mercurial inunction to any other means, and he employs it so vigorously that the greatest care is necessary to prevent ptyalism. Iodide of potassium is only recommended in those cases where there is a "depraved constitution,"—where the mercurial treatment does not produce the desired result, or when, in doubtful cases, (in the absence of alarming conditions) it is desired to establish the diagnosis by the treatment.

In these cases, he recommends this remedy in doses of from five to seven and a half grains every two hours.

The theory advanced, that paralysis of the recurrent laryngeal nerve, in cases of aneurisms and tumors, is due more to stretching than to pressure, would seem to be refuted by the alleged results of the treatment of sciatica by nerve stretching, as recommended by Prof. Nussbaum, of Munich.

In the treatment of paralysis of the laryngeal muscles, electricity is highly recommended, especially when employed through the pharynx. Strychnia, in solution, used hypodermically, is said to be "most worthy" of confidence. The dose recommended is at first gr. $\frac{1}{14}$, which in adults is soon increased to gr. $\frac{1}{7}$. These doses seem so large as not to be free from danger, yet the author claims to have used them "without detriment and with the best results."

Following this is an article by Steffen, on pseudo-croup, or "Spasm of the Glottis."

The article begins with a concise history of the disease, and the theories entertained by different authors as to its causation.

The author discards all others of the long list of names by which this affection has been designated, because, in his opinion, none of them so clearly represent the conditions of the disease as the one chosen.

According to the author, the paroxysm depends upon a spastic contraction of the muscles, which normally narrow the glottis, and the abnormal activity of these muscles has its foundation in a pathological irritation of the recurrent laryngeal nerve.

The predisposing cause of this spasm is, in nine cases out of ten, rachitis; and the principal exciting cause is disturbance of the rhythm of the respiratory acts. This disturbance is usually the result of suddenly awaking out of sleep, changes of position of the body, coughing, screaming, mental agitation, disturbances of mind, swallowing food, and abrupt changes of temperature.

In the etiology of this affection heredity has little, if any, influence. Catarrhs of the larynx, trachea and bronchi, are incapable of producing spasm of the glottis, and the thymus gland has nothing to do with this disease. Enlargements of the tracheal and bronchial glands, and some other conditions, are supposed to exert some influence in the causation.

In adults this disease cannot be caused by rachitis. It may result from local irritation of the larynx, or from reflex irritation, and as it occurs most frequently in females, these reflex manifestations usually have their basis in affections of the uterus.

Including all cases, the prognosis is thought to be, on the whole, favorable.

Regarding treatment during the paroxysm, the author suggests nothing new, but recommends those methods which, to us, seem least likely to afford prompt relief. Between the attacks his treatment usually resolves itself into combatting the real or fancied rachitis.

This volume contains much of interest to the general practitioner, which can hardly be ignored by those who have access to the writings of other authors upon the subjects of which it treats.

A Practical Treatise on the Diseases of Children. By *J. Forseith Meigs, M.D.*, and *William Pepper, A. M., M.D.* Sixth Edition. Revised and enlarged. *Lindsay & Blakiston*, Philadelphia. 1877.

More than any of the preceding, this edition assumes a place of high rank in the literature of Children's Diseases. In it there has been added an excellent article on cerebro-spinal meningitis, and a short one on night terrors; while that on cerebral congestion has been carefully re-written, and the subject presented in a much more comprehensive and satisfactory manner than heretofore.

These additions and alterations have been made in such a manner that the bulk of the volume is not materially increased. It seems to have been the endeavor of the authors to make this book complete and exhaustive, and at the same time to keep it within the limits of a text-book. The length, however, of the articles and the extent to which the opinions of other authors are given, render the discussions almost too extended for the student's limited time, and tend to make it more available as a book of reference. In fact, it is in this particular that the book is achieving its greatest success; and it bids fair to gain for itself, if it may not already be said to have gained, that place in this class of diseases, which Gross' surgery maintains in surgical literature. Its value would, we think, be greatly enhanced did it contain more particular information concerning gastric and intestinal hemorrhage of the new-born, opthalmia neonatorum, diseases of the umbilicus and umbilical hemorrhage—diseases for which we have looked in vain for satisfactory information.

We are also disappointed in finding no mention made of anodynes, narcotics, arterial sedatives, or of iodide of potassium, in the treatment of meningitis. Altogether the book is one which every practitioner, who aspires to excellence, should have in his library, and one which he cannot well afford to be without.
H. T. B.

Diseases of the Mind. Notes on the Early Management, European and American Progress, Modern Methods, etc., in the Treatment of Insanity. By *Chas. F. Folsom, M. D.*, Secretary Mass. Board of Health. *A. Williams & Co.*, Publishers, Boston.

The above monograph takes up, *seriatim*, the following subjects: Early Treatment of the Insane, Pinet's Reform and European Progress, English and American Modern Methods, Responsibility, Definitions, Massachusetts Statistics, State Supervision, Asylum Needs and Medical Education, treating them briefly from an enlightened stand-point, supporting the system of non-restraint, insisting upon special training for the care of the insane, and intelligent state supervision. Among the most interesting, is the sketch of Pinet's reforms in France, giving us a glance at the delusions which prevailed in regard to these unfortunates, and the barbarities practiced upon them, by both priest and physician, in the earlier ages. The book of Dr. Folsom is well worth a careful reading.
B.

The Practitioner's Reference Book. Adapted to the use of the Physician, The Pharmacist and The Student By *Richard J. Dunglison, M. D.* Philadelphia; Lindsay & Blakiston. 1877. Pp. 341. (Price, $3.50.)

A cursory view of the contents shows the great value of this book to the busy practitioner. The introduction is the Hipprocratic Oath. Following this, is a vast amount of "General Information for the Practitioner," embracing (1) weights and measures of the U. S. Pharmacopœia, (2) of the Metrical System, and of their inter-conversion; (3) number of drops in a fluid drachm ; (4) relative value of the drop and minim ; (5) approximative measurements ; (6) solubility of medicines ; (7) abbreviations and (8) comparison of thermometric scales. The work contains some therapeutic and practical hints for the sick-room, posology in its various aspects, directions for medicated baths, list of incompatibles, a condensed view of "the modern treatment of disease," indicating the diseases with the remedies therefor, (a section worth the price of the whole book), rules for the management of infants in the hot season, a tabular view of eruptive fevers, a diagnostic syllabus of uterine inflammations, obstetric memoranda embracing the best means of calculating the date of labor that we have seen, rules for urinalysis, for the treatment of cases of poisoning, for restoring the apparently drowned, for the application and use of disinfectants, and for preventing the spread of infectious diseases. The dietetic preparations and special forms of diet for the sick, and the rules for testing and disinfecting impure drinking-water, are compact, practical, and just what are not found in a similarly small space in any book published to-day. "How to conduct a post-mortem examination" is the most concise article in the work, and is contained in no general text-book obtainable in the United States.

The book is evidently compiled by a busy practitioner, prompted in his selection by a keen appreciation of the lack that all other busy practitioners experience in their libraries. A more judicious selection of subjects could not be made, and the class of physicians—the very busy ones—who need this book the most, will always feel that in purchasing this work they never before received so large a *quid pro quo*.

Principles of Theoretical Chemistry. By *Ira Remsen, M. D., Ph. D.*, Professor of Chemistry in the John Hopkins University. Philadelphia: *Henry C. Lea.* 1877.

As its title indicates this work confines itself exclusively to theoretical chemistry, and is, consequently, but little adapted to the uses of medical men. Those, however, who are desirous of informing themselves accurately concerning the beautiful theories and mathematical principles underlying our modern chemistry, cannot do better than to read Prof. Remsen's admirable book. The investigations of Dalton, of Gay Lussac, of Dulong, of Petit and of others, are carefully considered; Avogadro's law (which is playing such an important part in modern chemistry) is clearly explained, and the important deductions drawn from it, logically demonstrated; and the constitution of chemical compounds, as viewed in the light of our present accepted theories, receives somewhat extended consideration. The whole book is carefully written and fills the field that it is intended to occupy, more satisfactorily than any other similar work with which we are acquainted. W. S. H.

Chemia Coartata; or, The Key to Modern Chemistry. By *A. H. Hollmeyer, A. M., M. D.*, Professor of Materia Medica and Therapeutics at the University of Bishop's College; etc., etc. Philadelphia: Lindsay & Blakeston.

The arrangement of this work is decidedly novel, facts and principles being presented in the form of concise tables, so that a vast amount of information is contained in a comparatively small space. Owing to its tabular arrangement, it is especially useful as a book for rapid reference, and we believe that its author claims no more than is just, when he says, in the preface, that the work will be found "to be especially adapted to the wants of

1. Students intending to present themselves for examination;

2. Persons who have learned the *old* notation and wish to become acquainted with the *modern system.*

3- Those who desire to keep themselves posted on this subject, and who can thus easily refresh their memories without doing so at the expense of their other engagements.

W. S. H.

An Elementary Treatise on Practical Chemistry and Qualitative Inorganic Analysis. By *Frank Clowes, D.S.* London, Fellow of the Chemical Societies of London and Berlin, etc., etc. 1877.

Less comprehensive than Fresenius' classic, yet somewhat cumbersome, "Qualitative Analysis," but more extended than Elliot and Stone's popular manual, the above work seems to fill a want felt by many workers in analytical chemistry. The methods are modern, and the present approved system of nomenclature and notation are used exclusively,—facts which especially commend the book to new students in qulitative analysis. W. S. H.

